# Adapted Sequential Extraction Protocol to Study Mercury Speciation in Gold Mining Tailings: Implications for Environmental Contamination in the Amazon

**DOI:** 10.3390/toxics12050326

**Published:** 2024-04-30

**Authors:** João Pedro Rudrigues de Souza, Jeremie Garnier, Julia Mançano Quintarelli, Myller de Sousa Tonhá, Henrique Llacer Roig, Patrick Seyler, Jurandir Rodrigues de Souza

**Affiliations:** 1 Institute of Chemistry, University of Brasília, Asa Norte, Brasilia 70910-900, Brazil; rodsouza@unb.br; 2 Institute of Geosciences, University of Brasília, Asa Norte, Brasilia 70910-900, Brazil; garnier@unb.br (J.G.); myllerquimico@gmail.com (M.d.S.T.); roig@unb.br (H.L.R.); 3 HydroSciences Montpellier, Université de Montpellier, Institut de Recherche Our le Développement, Centre National de la Recherche Scientifique, 34090 Montpellier, France; patrick.seyler@ird.fr

**Keywords:** mercury, speciation, ASGM, tailings, sequential extraction procedure, Amazon

## Abstract

Artisanal small-scale gold mining (ASGM), an increasingly prevalent activity in South America, generates mercury-contaminated tailings that are often disposed of in the environment, leading to the introduction of mercury into ecosystems and the food web, where it bioaccumulates. Therefore, studying the geochemical processes involved in the desorption and dissolution of mercury in these tailings is essential for critical risk evaluations in the short and long term. For this purpose, sequential extraction procedures (SEPs) can be useful because they help to identify the phases to which Hg is associated, although they also have limitations such as a lack of selectivity and specificity. In this work, we propose a modified four-step SEP: exchangeable mercury (F1), oxidizable mercury (F2), mercury bound to Fe oxides (F3), and strongly bound mercury (F4). To test this adapted sequential extraction method, we evaluated the Hg contamination in mercury-contaminated tailings of the Amazon basin. The results revealed a total mercury concentration of 103 ± 16 mg·kg^−1^ in the tailings, with a significant portion in F1 (28% of the total), where Hg was bioavailable. The large Hg concentration in F3 (36%) suggested that Fe oxides likely contribute to mercury retention. Together, the SEP results emphasize the urgent need for improved surveillance of gold mining activities and responsible tailings management practices to mitigate environmental contamination and safeguard the health of the Amazon ecosystem.

## 1. Introduction

The Amazon basin is regularly highlighted for environmental and human health issues stemming from various human activities. Deforestation or rainforest fires [1] and mercury (Hg) pollution [2], associated with gold mining [3], play a central role in this concern. Over the past two decades, due to the increase in gold prices, mercury pollution resulting from illegal gold mining in South American countries has surged, emerging as the primary cause of substantial emissions of Hg vapor into the atmosphere and waterways [4,5,6]. In the Amazon, gold occurs at low concentrations in soils and riverbeds, where artisanal small-scale gold mining (ASGM) produces mercury-contaminated tailings that are irresponsibly disposed of [7]. Practices vary, but miners typically use heavy machinery to excavate huge amounts of material or hoses to suck up sediment from the riverbed and then process these sediments through a gravimetric separation system (carpets) that concentrates the heavy minerals, including gold. The miners then add liquid mercury to a slurry of gold-containing sediments so that Hg bonds with the gold, forming an amalgam. Miners discard the process water and tailings, which still contain some mercury, and then heat the amalgam, vaporizing the mercury and leaving behind solid gold. In summary, the absence of a well-defined ASGM waste management protocol results in the indiscriminate dumping of Hg into rivers [8,9], elevating the risk of methylmercury (MeHg) production—a toxic species generated by bacteria present in river sediments [10].

Regarding Hg, more than its total concentration in sediments, its toxicity and risk to human health [11,12] are closely tied to its speciation [13]. In sediments, inorganic Hg can be bound to both mineral phases and organic matter (OM) [14]. However, in solid materials with low OM contents, such as industrial and mining tailings, Hg is primarily associated with Fe and Mn oxyhydroxides [15]. Altogether, the distribution of mercury in various bearing phases depends on variables such as particle size [16], the surface area of the minerals, the pH of the medium, and the presence of ligands such as Cl^−^ and SO_4_^2−^, along with other organic molecules [14].

Therefore, studying the speciation and availability of mercury in contaminated sediments such as mining tailings is essential for assessing the environmental and occupational risks associated with ASGM activities involving this element. As an example, ASGM impacts on the Madeira River Basin have been studied for years by determining total mercury (THg) and MeHg in sediments, soils, water, particulate matter, aquatic biota, and humans [17,18,19,20,21]. Yet, there is a lack of studies on Hg speciation on tailings from ASGM dredges, which is arguably the most intense form of gold mining in this basin [8].

Among the techniques for Hg speciation, X-ray absorption spectroscopy (XAS) provides higher specificity, offering information about the oxidation state and the neighboring atoms of mercury [22,23]. However, its accessibility is limited due to the high cost of equipment and the complex infrastructure required for analysis. As an alternative, sequential extraction is a tool that allows for the separation of mercury fractions through a simpler and lower-cost procedure, providing information on interaction with the matrix, solubility, and mobility [24,25,26].

The most widely used sequential extraction procedure (SEP) for mercury in the literature is that of Bloom et al. (2003), which consists of a five-step extraction: F1 (deionized water), F2 (0.01 mol·L^−1^ HCl + 0.1 mol·L^−1^ CH_3_COOH), F3 (1 mol·L^−1^ KOH), F4 (12 mol·L^−1^ HNO_3_), and F5 (*aqua regia*) [27]. Despite its broad applicability, this protocol has limitations such as a lack of specificity and selectivity [28,29], low recoveries for some samples [30], and a long experiment time. Therefore, an adaptation of the methodology becomes necessary to address these issues.

Motivated by these challenges, in this study, we propose a new adapted SEP to investigate the speciation of mercury in ASGM dredge tailings. Additionally, Hg-bearing phase characterization was investigated using mineralogy and geochemistry approaches. The results provide complementary information to define each extraction step fraction. The approach was applied to tailings from a Madeira River ASGM dredge, given the significant number of ASGM dredges on its course [8] and the issue surrounding Hg in the Upper Madeira River Basin. Our findings allowed us to infer the environmental fate and mobility of mercury associated with the discharge of such highly contaminated ASGM tailings into an Amazonian ecosystem. Furthermore, our dataset allows for an environmental risk discussion considering its interaction with the native matrix under aqueous conditions and dispersion in downstream ecosystems.

## 2. Materials and Methods

### 2.1. Sampling Site

The Madeira River has a water mass perimeter of 2752.39 km, with an area of 1566.03 km². It is the second-largest tributary of the Amazon River and contributes to approximately 50% of its sediment load [31]. It originates in the confluence of the Mamoré and Beni Rivers and is characterized as a whitewater river with a large abundance of suspended material and a pH between 6 and 7. The suspended sediment load presents a geological composition characteristic of the Andean regions (sedimentary and volcanic rocks) and the Central Brazil Shield (igneous and metamorphic rocks) [32,33].

### 2.2. Sampling and Sample Preparation

All materials used in collection, storage, and analysis underwent cleaning in a 5% HNO_3_ bath for at least 24 h, followed by triple rinsing with deionized water (18.0 ± 0.2 MΩ·cm^−1^).

An ASGM tailings sample was collected from a mining dredge operating in the Madeira River (−9.586 latitude, −64.927 longitude) in April 2022. The tailings sample was carefully taken, with a plastic bag, in a tank designated for storing the material to be disposed of after the addition of mercury for gold amalgamation. The tailings were dried in an oven at 40 °C and ground in an agate mortar before analysis to ensure the homogeneity of the sample. 

### 2.3. Mineralogy and Chemical Composition

The mineralogical composition of river sediments, tailings, and residues from each stage of the sequential extraction was determined in the <63 μm fraction by X-ray diffraction (XRD) using the Rigaku^®^ Ultima IV diffractometer with Cu Kα radiation (λ = 1.5406 Å), operating at 45 kV and 15 mA. The 2θ range was from 2° to 60° with a scan rate of 0.083°/1 s. Mineral identification was based on their characteristic peaks using MDI Jade^®^ software version 9.

We used scanning electron microscopy coupled with energy dispersive X-ray spectroscopy (SEM-EDS) to identify the chemical composition at a microscopic level. For this, a portion of the sample was deposited on a double-sided tape and analyzed, without metallization, on a Jeol JSM-7000F with an accelerating voltage of 15 kV.

The procedure to determine organic matter content by loss of ignition (LOI) was, briefly, to weigh 2.0 g of dry sediment in a porcelain crucible and insert it into an oven at 450 °C for 4 h. The mass lost during the heating process could be considered an approximation of the organic matter mass of the sample.

### 2.4. Hg Quantification

Mercury determination was performed by atomic absorption spectroscopy with Zeeman correction (ZAAS RA-915+^®^, Lumex Instuments, St. Petersburg, Russia). For solution analysis, we utilized the RP-91 accessory to perform cold vapor atomic absorption spectroscopy (CVAAS), with an airflow (ambient air) of 1.2 L·min^−1^, sample volume of 0.05 mL, blank volume of 0.95 mL, 10% SnCl_2_ solution volume (reducing agent) of 0.4 mL, and 1% NaCl solution volume of 10 mL. All solutions were analyzed on the same day of extraction to prevent analyte loss. For solid sample analysis, the PYRO pyrolysis furnace accessory was used, which allowed for solid sample analysis by thermal desorption atomic absorption spectroscopy (TDAAS). The operating mode used had an airflow (ambient air) of 1.2 to 0.8 L·min^−1^, first atomizer chamber temperature of 680 to 740 °C, second atomizer chamber temperature of 600 to 770 °C, and analytical cell temperature of 680 to 730 °C.

The analytical curves for the CVAAS mode were prepared from a standard solution of 1000 µg·mL^−1^ Hg in 15% HNO_3_ Fluka^®^( Charlotte, NC, USA). Dilutions were made in a solution that simulated the extract’s blank, composed of nanopure water, BrCl, and the solution used for extraction in each step of the procedure. The limits of determination (LD = 10 × SD/b) calculation was based on the standard deviation (SD) of six measurements of the extract’s blank and each analytical curve’s angular coefficient (b) (Table S1). Accuracy was determined by the analysis of an Aldrich Hg standard solution, with a recovery of >80%.

To determine total mercury in solid samples in TDAAS mode, the analytical curve (peak area versus Hg mass) was made by weighing different portions of the PACS-3 reference material (marine sediment). The tailings bulk sample was mixed with silica gel for chromatography (0.05–0.20 nm) Carlo Erba^®^ (Emmendingen, Germany) and homogenized in an agate mortar and pestle before analysis. The limit of determination (LD = 10 × SD/b) calculation was based on the standard deviation (SD) of seven blank measurements and the analytical curve’s angular coefficient (b) (Table S1). Accuracy was determined by analyzing the NIST 1646a reference material (estuarine sediment), which showed a recovery of 94 to 105%.

The precision for all mercury determinations was verified by the relative standard deviation (RSD) of triplicate measurements. All triplicates had RSD < 13%.

### 2.5. Hg Sequential Extraction Procedure

The sequential extraction procedure was based on the original methodology of Bloom et al. (2003), which consists of a five-step extraction of Hg [27]. Adaptations from Hall et al. (2005) and Pinedo-Hernandez et al. (2015) [34,35] were also incorporated. Additionally, an extraction step with 6 mol·L^−1^ HCl was added, aiming to remove Hg bound to Fe oxides and hydroxides, following Vasques et al. (2020) [29], and the removal of the first (deionized water) and third (1 mol·L^−1^ KOH) steps of the original procedure, to simplify the experiment and because the samples used did not contain significant levels of organic matter to justify the use of the KOH step. A final modification was made by replacing the acid decomposition of the sample in the last step with the determination of residual Hg in the solid residue of the extraction by TDAAS. Thus, after removing the supernatant with the third fraction, the solid residue was washed with MiIliQ water, dried in an oven at 40 °C, and taken for TDAAS analysis. The applied procedure is detailed in Table 1 in instructional form. The accuracy of the whole procedure was evaluated by the recovery relative to the THg in the tailings sample, which varied from 89% to 101%.

### 2.6. Major Elements Determination

Major elements (except for K and Na) in the tailings were quantified at the Geochemistry Laboratory, University of Brasília, by ICP-OES (Agilent 5100) after alkaline fusion with lithium metaborate in a furnace at 950 °C for 60 min, followed by dissolution in 2 mol·L^−1^ HCl. K and Na were determined by ICP-MS (Thermo Scientific^®^ ICAP-Q at Hydrosciences Montpellier Laboratory, Paris, France) after a 4-step total acid digestion procedure in a Savillex^®^ PFA reactor(Eden Prairie, MN, USA) [36]. Major elements in the extraction solutions were also analyzed by ICP-OES after dilution with deionized water. Certified reference material NIST 1646a was used for analytical quality assurance in all analyses and recoveries for each element were between 86% and 98%. Precision was verified by the RSD of triplicate measurements. All triplicates had RSD < 10%.

### 2.7. Software and Data Analysis

Microsoft Excel 365 was used to process all numerical data and calculate uncertainties for the results, as well as to plot the Hg SEP results and XDR results, which were exported from MDI Jade^®^ version 9 (Bodie, CA, USA). Additionally, GraphPad Prism 8.0 was used for plotting elemental concentrations in the sequential extraction solutions and performing the Grubbs test to identify outliers (alpha = 0.05). However, no outliers were identified in our datasets. The measurements’ uncertainties are expressed as confidence intervals at a 95% confidence level, which were calculated by multiplying the propagated uncertainty of the measurements by the adequate Student’s t value and dividing by the square root of the number of replicates (n).

## 3. Results and Discussion

### 3.1. Geochemical Characterization of the Tailing

No organic matter was detected in the tailings, and minerals identified by XRD in the tailings included quartz (SiO_2_, density = 2.62 g·cm^−3^), zircon (ZrSiO_4_, density = 4.65 g·cm^−3^), ilmenite (FeTiO_3_, density = 4.72 g·cm^−3^), magnetite (Fe_3_O_4_, density = 5.15 g·cm^−3^), and hematite (Fe_2_O_3_, density = 5.30 g·cm^−3^). Additionally, in some points analyzed by SEM-EDS, minerals with chemical compositions similar to monazite ((Ce,La,Nd,Th)PO_4_, density = 5.15 g·cm^−3^) were found (Table S2). Except for quartz, these minerals are considered heavy due to their densities above 2.87 g·cm^−3^, and they reflect the gravimetric separation process through which the sediments pass in the mining dredge [37].

THg concentration in the tailings was 103 ± 16 mg·kg^−1^ (n = 6), which exceeded by 10,300 times the background value of 0.01 mg·kg^−1^ for the Madeira River sediments defined by Pfeiffer [17] and was above values found in other studies [38,39,40,41] (Table 2). The chemical composition of elements conventionally characterized as major was 21.56% Fe, 12.71% Si, 6.33% Ti, 0.81% Al, 0.60% P, 0.32% Mn, 0.21% Ca, 0.16% Mg, 0.53% K, and 0.07% Na. Additionally, specifically in the case of this sample, Zr was considered a major element, with a concentration of 13.92%.

Considering the high affinity of Hg for negatively charged surfaces [42], the high value of THg retained in the tailings may be related to the presence of Fe oxides that exhibit a negative charge at a neutral pH [43,44].

### 3.2. Changes in Mineralogy during the Sequential Extraction Procedure (SEP)

**Figure 1:**
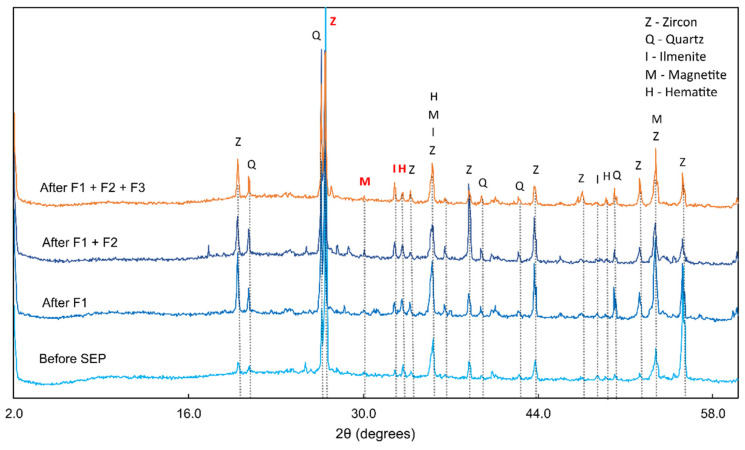
X-ray diffractogram of tailings sample before and after each step of the SEP. The peaks cited in the discussion are represented in red letters: d = 2.70 Å (hematite), d = 2.76 Å (ilmenite), d = 2.97 Å (magnetite), and d = 3.31 Å (zircon).

To assess the efficiency of each extraction step, we proposed a semi-quantitative approach for evaluating the dissolution of Fe oxides. This method involved comparing the X-ray diffraction (XRD) patterns between two consecutive SEP steps. To accomplish this, we utilized zircon as a reference because it is a mineral considered extremely resistant to acid dissolution. Therefore, the intensity of zircon’s highest peak, at interplanar distance (d) = 3.31 Å, was compared with the intensity of peaks from hematite (d = 2.70 Å), magnetite (d = 2.97 Å), and ilmenite (d = 2.76 Å) (refer to Table 3). Following F1, there was an observed increase in the contribution of zircon, hematite, magnetite, and ilmenite to the overall mineral composition of the sample. This can be attributed to the dissolution of residues from more soluble minerals that were not identifiable in the diffractogram (see Figure 1). 

After F2, the ratios of the magnetite and ilmenite increased relative to zircon (refer to Table 3), suggesting the preservation of these oxides in the sample. The ratio of the hematite peak remained constant, indicating no significant alteration in these minerals after HNO_3_ treatment.

Following F3, a decrease in the ratios of the hematite and magnetite peaks relative to zircon was observed, indicating that HCl partially dissolved the target minerals of the fraction (iron oxides), as the peaks of these minerals were still present in the diffractogram. Inversely, the ratio of the ilmenite peak relative to zircon increased, revealing the mineral’s resistance to 6 mol·L^−1^ HCl.

### 3.3. Changes in Chemistry during the SEP

**Figure 2:**
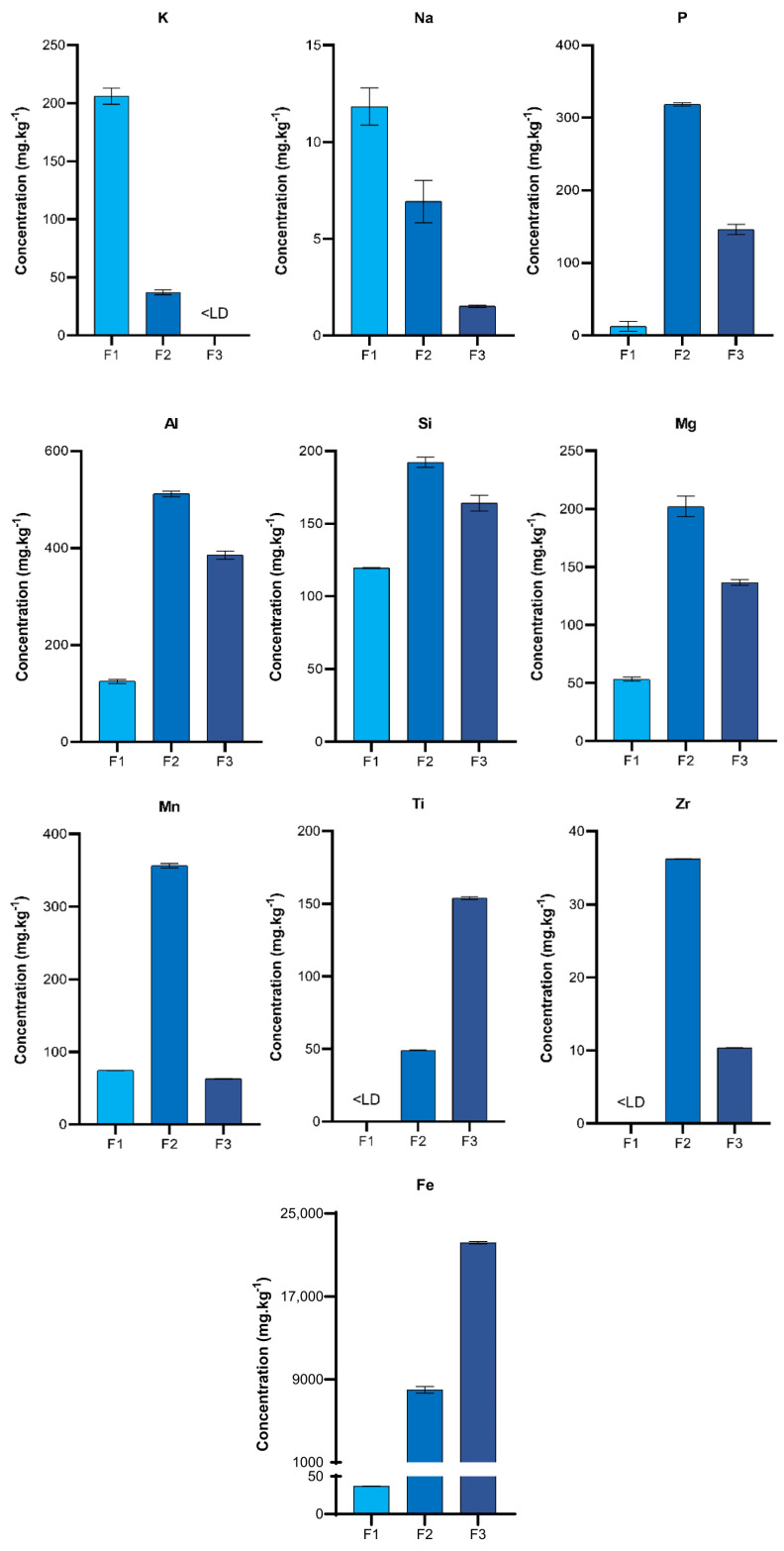
Graphs of concentrations of the elements analyzed in the sequential extraction solutions. Error bars represent the confidence intervals at the 95% confidence level (n = 3).

Validation was not conducted for the extraction of elements other than Hg in the solutions as more suitable SEP protocols, such as the Community Bureau of Reference (BCR) and Tessier methods, exist for them. However, a semi-quantitative analysis of the extraction profile (Figure 2) of these elements was performed to complement the information on the compounds dissolved during the extractions (Table S4).

The low concentrations of extracted Zr (Figure 2) demonstrated a high resistance of zircon to the extracting solutions, considering it was one of the most abundant minerals in the tailings, according to XRD analysis (Figure 1), and that Zr constituted 13.92% of the sample. Similarly, the amount of Si extracted (Figure 2) did not reflect the significant contribution of quartz peaks in the diffractogram (Figure 1), confirming that quartz was insoluble in any of the reagents used, as expected. Furthermore, despite the significant Si concentrations in the extraction profile (Figure 2), the sum was still small when compared to that of the total sample (0.4% of total Si). Therefore, it can be assumed that the extracted Si originated from other less resistant and less abundant silicates in the sample such as illite, chlorite, kaolinite, and smectite, which did not appear in the diffractograms, but are typical clay minerals from the Madeira River basin [45].

K and Na had the highest concentrations in F1 (Figure 2), indicating that the solution could indeed dissolve most of the elements that participated in weak electrostatic interactions, were known to be bound to negatively charged surfaces of minerals, and were released in the process of cation exchange with H^+^ from the acids [42].

In F2, Al, Mg, Mn, P, Si, and Zr exhibited their highest concentrations (Figure 2), indicating a higher susceptibility of these elements to oxidation promoted by HNO_3_ and the probable partial dissolution of clay minerals and Al and Mn oxyhydroxides.

In F3, Fe and Ti presented their highest concentrations (Figure 2). We hypothesized that the majority of the extracted Fe did not originate from the iron oxides observed in the XRD analysis as their peaks did not disappear after the F3 extraction but rather from weathered (or secondary) Fe oxyhydroxides. Although not identified in the diffractograms, weathered minerals may have existed in smaller proportions (<2%) and contributed to the baseline noise observed in Figure 1. These secondary minerals, such as Fe oxyhydroxides, are known to sequester elements such as Ti [42] and could have contributed to the concentrations of Ti extracted in F3.

Finally, based on changes in mineralogy and element extraction profiles, we named the fractions (or the extraction steps) as follows:

- F1: exchangeable mercury—weakly bound to the sample matrix and, therefore, more bioavailable. Extraction accompanies the dissolution of highly soluble minerals and elements known for cation exchange.
- F2: oxidizable mercury—poorly soluble and extracted through the oxidation of bonds with HNO_3_. Extraction accompanies the dissolution of Fe, Al, and Mn oxyhydroxides.
- F3: mercury bound to Fe oxides—poorly soluble and primarily associated with Fe oxides. Extraction accompanies the dissolution of Fe, Al, and Mn oxyhydroxides, as well as the partial dissolution of magnetite and hematite.
- F4: strongly bound mercury—forming highly stable bonds to minerals in the sample matrix. Possibly strongly bound to zircon, ilmenite, hematite, and magnetite or included in the internal structure of minerals.

### 3.4. Hg Speciation

**Figure 3:**
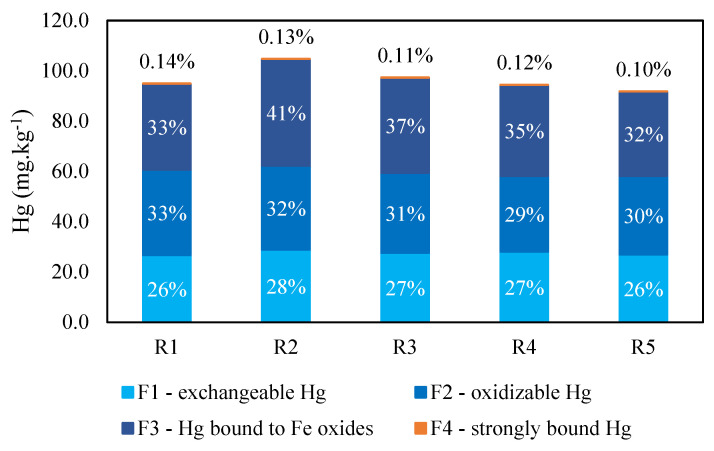
Graph with Hg content in each SEP fraction. The colors represent different fractions, and values within each bar, or at the top, in the case of the residue, are the percentages of each fraction relative to THg.

Figure 3 and Table 4 present the mercury contents found in each fraction and recovery relative to the total Hg concentration in the sample before extraction. The average recovery (ΣFn/THg) was high at 94%. The fraction with the highest Hg content was F3, with an average of 36.9 ± 3.6 mg·kg^−1^ and a proportion of 36% of THg. This indicates a preferential interaction of mercury with Fe oxides. This interaction has been observed in previous studies [15,46], and Biester et al. (2002) reported that Hg bound to Fe oxides and hydroxides in organic-poor soils near a chlor–alkali industry [47].

After F3, the fractions with the highest Hg contents were F2 and F1, comprising 31% and 27% of the total, respectively. Although the Hg from F1, weakly bound and more available, constituted a lower proportion in the tailings than F2 and F3, the average concentration of 27.6 ± 0.9 mg·kg^−1^ found in this fraction already indicated a severe contamination risk for the biota of the Madeira River. This value far surpassed the background level of 0.01 mg·kg^−1^ and, being the most bioavailable fraction, underlines the harmfulness of tailings for the ecosystem and human health, as discussed further in the *Environmental implications* section.

The original work by Bloom attributed elemental mercury to the fraction soluble in HNO_3_ (F2 in this work) [27]. However, this species is scarcely found in samples stored for more than ten days [48]. Furthermore, studies that identified mercury in this fraction and conducted speciation assays by TDAAS emphasize that Hg(0) is not a relevant species in the samples’ composition. The species present in this fraction can be Hg(II) bound to amorphous organosulfides and Fe and Mn oxyhydroxides [15,49]. Gilli et al. (2018) even observed an increase in the concentration of Fe accompanying the increase in mercury in the fraction soluble in HNO_3_ [49]. Therefore, we can infer that while part of the Hg(0) added to the sediment evaporates locally during the mining process, another part oxidizes to Hg(II) during the brief time that it is stored in tanks inside the dredges. Due to the stability of Hg(0) regarding the oxidation process, the substantial amount of mercury added to amalgamate with the gold already leads to high concentrations of Hg(II), even though only a minor fraction would be oxidized.

To compare, Magalhães and Tubino (1995) showed that Hg added in drops to a NaCl 30 G·L^−1^ solution under agitation oxidized and reached a concentration of 13 µg·mL^−1^ in less than 3 h of the experiment [50]. Additionally, another study on areas contaminated by chlor–alkali industries indicates that, over time, Hg(0) oxidizes and forms recalcitrant compounds with sulfur (HgS, Hg_3_S_2_Cl_2_) or more soluble compounds such as HgSO_4_ and HgO [24]. Since sulfur was not detected in our sample, it is expected that, before coming into contact with river sediments, Hg is already oxidized and interacting with other elements. However, once in an environment with sulfur atoms and organic matter, the formation of compounds with Hg-S bonds and Hg-OM complexes can occur.

The low content in F4, averaging 0.1% of the total or 0.13 ± 0.02 mg·kg^−1^, indicates that the strongly bound mercury contributes minimally to the total in the tailings. This may suggest that it comprises mercury that is naturally present in the heavy minerals of the Madeira River sediments and is, thus, immobilized. This finding agrees with the larger content of Hg in mined sediments being from an anthropogenic source, similar to the findings of Goix et al. (2019) using mercury isotopic signatures [51].

### 3.5. Environmental Implications

While the majority of the Hg in the tailings was present in fractions suggesting low mobility (poorly soluble), the average value of 27.6 ± 0.9 mg·kg^−1^ in F1 (exchangeable) was already more than 2700 times higher than the background value for sediments in the Madeira River (Table 2) [17]. The unregulated disposal of this material into the river, along with the continuous dredging of sediments, leads to sediment resuspension in the water column. This facilitates the transport of contaminated tailings along the river course through suspended solids and leads to the dispersion of large Hg amounts. Additionally, reintroducing mercury-rich tailings with a large bioavailable pool into the river may result in interactions with other mineral phases and biogeochemical processes, altering its mobility, bioavailability, and bioaccessibility in the natural environment. 

The portion in the exchangeable fraction (F1) may contribute to the flux of Hg in the water, where it can become associated with biofilms, which serve as compartments for Hg entry into the food chain [52]. This happens because Hg in this fraction is bonded to the matrix by weak electrostatic interactions that can be easily broken down by cation exchange processes [53]. When mercury is desorbed from the matrix in the river, it can be released to the dissolved phase and re-adsorbed in the particulate phase, forming more stable bonds with the S atoms from the OM [54]. Then, once Hg is associated with OM, it can be transported through greater distances than it could when it was adsorbed in the tailings, increasing the reach of the contamination.

F2 (oxidizable) and F3 (bound to Fe oxides), together, comprised 67% (69 mg·kg^−1^) of the total mercury in the tailings and were defined as poorly soluble fractions. Despite this, they could also be considered pollution sources depending on the pH and redox potential of the environment in which they are introduced. For instance, Liang et al. (2019) found that in environments with a pH ranging from 4.33 to 7.07, mercury in tailings discarded in the river can migrate, over time, from the fraction soluble in HNO_3_ to one soluble in KOH (associated with organic matter) [55]. This suggests that in whitewater rivers, such as the Madeira River, with a pH range of 6 to 8.5 [56], Hg species redistribution could occur in situations where these pH ranges overlap.

Furthermore, our results indicate a severe risk of Hg species redistribution in ASGM tailings samples from the Madeira River. Following the transport of tailings particles, Hg in F1, F2, and F3 (94% of THg, 96.6 mg·kg^−1^) may eventually transform into MeHg in the sediment if it reaches environments such as floodplains, estimated to be 800.000 km^2^ in the Amazon basin [57], and water reservoirs associated with hydroelectric power plant dams [58,59]. Their sediment, rich in clay fraction and OM, can provide adequate conditions for methylation, such as low oxygen levels at the bottom of the water column and reducing conditions in surficial sediment, in which sulfate-reducing and iron-reducing bacteria take in Hg(II) from sediments and produce MeHg [60,61,62]. Previous studies have shown that reservoirs, such as the one at the Jirau hydroelectric power plant, downstream from the dredge where the tailings were sampled, not only are responsible for increased MeHg production locally but can impact downstream areas as well [60].

In a scenario where the studied tailings reach a floodplain, where changes in redox potential occur seasonally [63], Hg in F2 and F3 could change its speciation and become more labile, leading to it being more easily redistributed to mineral and organic phases in bottom sediments and particulate matter. This would make it available for methylation, such as was previously observed by Pestana et al. (2019) in the Cuniã Lake, a floodplain in the Madeira River Basin, where the authors also demonstrated Hg transfer from sediment to biota [19].

Finally, it is also worth pointing out that Crowther et al. (2021) reported the dissolution of strongly bound Hg compounds in sediments from an area impacted with Hg(0) and emphasized that Hg soluble in HNO_3_ or aqua regia can be dissolved in water and re-adsorbed onto sediments as compounds with higher mobility than they originally had [52]. This implies that even F4 could be a source of Hg for the methylation process in the long term.

## 4. Conclusions

The adapted and applied sequential extraction procedure (SEP) seems particularly well suited to the separation of distinct mercury fractions from the ASGM tailings samples derived from dredge operations in rivers. In the Madeira River sample, the SEP was applied with high recovery rates (89% to 101%), taking into consideration the mineralogy and chemical composition of the tailings before the analysis. The total mercury concentration was notably high at 103 ± 16 mg·kg^−1^, significantly surpassing the background level. The SEP results indicate that Hg preferentially binds to Fe (hydr)oxides. 

The geochemical characterization and mercury speciation of the tailing samples unveil a severe contamination risk to the Madeira River ecosystem, seeing that there was 27% of THg in the most bioavailable fraction. Furthermore, the disposal of tailings into rivers poses both immediate and long-term risks, considering potential Hg redistributions in aquatic ecosystems downstream of tailing disposal sites. Considering the Hg speciation results, the high concentration of Hg in a weakly bound form (27.6 mg·kg^−1^) in the tailings can facilitate methylmercury production in reducing conditions during post-depositional processes, while other mercury fractions are more likely to represent a long-term Hg source for the aquatic ecosystem.

In conclusion, this study emphasizes the imperative need for gold mining surveillance in the Amazon and advocates for responsible tailing management practices to mitigate environmental contamination and safeguard the health of the ecosystem. Additionally, we encourage future Hg speciation studies in other ASGM tailings in highly impacted rivers such as Beni and Oiapoque and indigenous territories like Yanomami and Mundurucu.

## Figures and Tables

**Table 1:** Adapted sequential extraction procedure for mercury speciation in gold mining tailings.

Fraction Number	Extracting Procedure	Fraction Name
1	Weigh 0.5 g of dry sediment into a 40 mL centrifuge tube and add 6.25 mL of 0.1 mol·L^−1^ HAc + 0.01 mol·L^−1^ HCl. Leave agitating for 18 h ± 4 h. Centrifuge at 3000 rpm for 20 min. Pour off the supernatant solution (aliquot 1) into another tube. Add 0.25 mL of 0.2 mol·L^−1^ BrCl to aliquot 1. Rinse the residue (add another 6.25 mL of the same extracting solution and shake vigorously). The rinsed aliquot should be added to the first. Analyze the solution via CVAAS immediately after completing the extraction procedure.	Exchangeable mercury—soluble in 0.1 mol·L^−1^ HAc + 0.01 mol·L^−1^ HCl
2	In a fume hood, add 6.25 mL of 6 mol·L^−1^ HNO_3_ to the residue from step 1. Leave agitating for 18 h ± 4 h. Centrifuge at 3000 rpm for 20 min. Pour off the supernatant solution (aliquot 1) into another tube. Add 0.25 mL of 0.2 mol·L^−1^ BrCl to aliquot 1. Rinse the residue (add another 6.25 mL of the same extracting solution and shake vigorously). The rinsed aliquot should be added to the first. Analyze the solution via CVAAS immediately after completing the extraction procedure.	Oxidizable mercury—soluble in 6 mol·L^−1^ HNO_3_
3	In a fume hood, add 6.25 mL of 6 mol·L^−1^ HCl to the residue from step 2. Leave agitating for 18 h ± 4 h. Centrifuge at 3000 rpm for 20 min. Pour off the supernatant solution (aliquot 1) into another tube. Add 0.25 mL of 0.2 mol·L^−1^ BrCl to aliquot 1. Rinse the residue (add another 6.25 mL of the same extracting solution and shake vigorously). The rinsed aliquot should be added to the first. Analyze the solution via CVAAS immediately after completing the extraction procedure.	Mercury-bound Fe oxides—soluble in 6 mol·L^−1^ HCl
4	Rinse the residue from step 3 with ultrapure water: add approximately 10 mL of water to the tube with the residue, shake manually, centrifuge at 3000 rpm for 10 min, and discard the supernatant. Repeat this step 3 times and, finally, dry the residue in an oven (40 °C). Analyze the dried solid residue by TDAAS.	Residual or strongly bound mercury

**Table 2:** Total mercury concentrations in the tailing sample of this study and those of other studies performed on gold mining tailings and the sediment background value for the Madeira River.

	THg (mg·kg^−1^)
Background for Madeira River sediments [17]	0.01
Odumo et al., 2014 [38]	8.90 ± 2.56 (SD, n = 41)
de Andrade Lima et al., 2008 [39]	6.55 ± 3.80 (SD, n = 7)
Tibane and Mamba, 2023 [40]	0.63 ± 0.99 (SD, n = 10)
Opiso et al., 2018 [41]	0.3 to 25.02
This work	103 ± 16 (CI_95%_, n = 6)

Background value for Madeira River sediments described as the natural level by Pfeiffer et al. (1991) [17]. SD—standard deviation. CI_95%_—confidence interval at 95% confidence level.

**Table 3:** Intensities for characteristic peaks of hematite, ilmenite, and magnetite divided by the intensity of zircon’s peak at d = 3.31 Å.

	Hematite	Ilmenite	Magnetite
	Ratio d_2.70_/d_3.31_	Ratio d_2.76_/d_3.31_	Ratio d_2.97_/d_3.31_
Before SEP	0.02	0.01	- ^a^
After F1	0.07	0.05	0.02
After F2	0.07	0.08	0.04
After F3	0.05	0.12	0.03

The ratios represent each mineral’s contribution relative to zircon, which was chosen as the denominator due to its conservative behavior in the sample. ^a^—magnetite could not be identified before the SEP.

**Table 4:** Mercury concentrations and percentages determined in each fraction through the SEP in five laboratory replicates.

Replicates	F1		F2		F3		F4		ΣFn	Recovery
	mg·kg^−1^	F1/THg	mg·kg^−1^	F2/THg	mg·kg^−1^	F3/THg	mg·kg^−1^	F4/THg	mg·kg^−1^	
R1	26.66 ± 0.04	26%	34.0 ± 0.1	33%	34.2 ± 0.3	33%	0.15 ± 0.02	0.1%	95.0 ± 0.3	92%
R2	28.76 ± 0.04	28%	33.4 ± 0.1	32%	42.5 ± 0.4	41%	0.132 ± 0.007	0.1%	104.8 ± 0.4	101%
R3	27.52 ± 0.04	27%	31.8 ± 0.1	31%	37.9 ± 0.3	37%	0.12 ± 0.01	0.1%	97.4 ± 0.4	94%
R4	28.03 ± 0.04	27%	30.1 ± 0.1	29%	36.2 ± 0.3	35%	0.13 ± 0.02	0.1%	94.5 ± 0.3	91%
R5	26.88 ± 0.04	26%	31.3 ± 0.1	30%	33.6 ± 0.3	32%	0.10 ± 0.02	0.1%	91.8 ± 0.3	89%
Average	27.6	27%	32.1	31%	36.9	36%	0.13	0.1%	96.7	94%
RSD	3%	3%	5%	5%	10%	10%	14%	14%	5%	5%

ΣFn represents the sum of the mercury in all four fractions and the recovery is expressed relative to the total mercury in the tailing (THg = 103 ± 16 mg·kg^−1^).

## Data Availability

All data used in this work is available either within the article or in the supporting information file.

## Supplementary Materials

The following supporting information can be downloaded at: https://www.mdpi.com/article/10.3390/toxics12050326/s1, Figure S1: SEM image of the tailings sample with targeted spots represented in points where EDS analysis was performed; Table S1: Analytical parameters of mercury determination; Table S2: EDS results in % weight of each element; Table S3: Major elements concentration in the ASGM tailings sample; Table S4: Elements concentration in the first three sequential extraction procedure steps.
