# Supplementary material for: Adapted Sequential Extraction Protocol to Study Mercury Speciation in Gold Mining Tailings: Implications for Environmental Contamination in the Amazon
Source: Toxics. 2024 Apr 30;12(5):326. doi: 10.3390/toxics12050326 (PMC11125949; doi:10.3390/toxics12050326)
Supplement: Supplementary file 1 [file toxics-12-00326-s001.zip › toxics-2938464-supplementary.pdf]

## Supplementary figures and tables

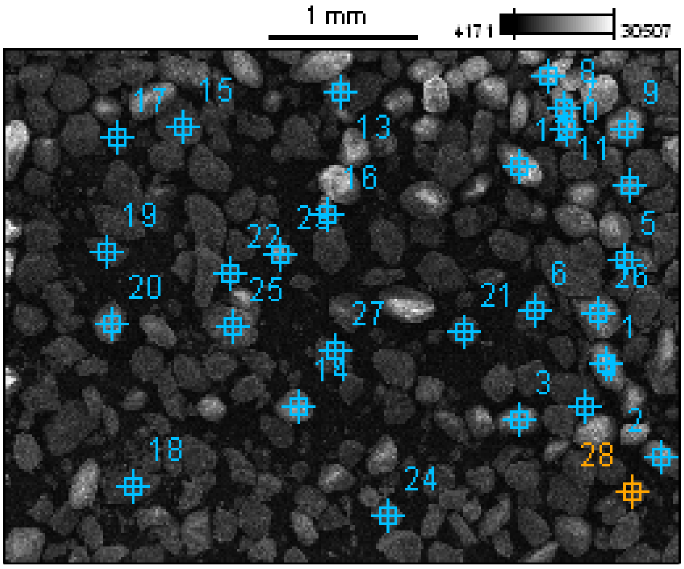


**Figure S1** – SEM image of the tailings sample with targeted spots represented in points where EDS analysis was performed.

**Table S1** - Analytical parameters of mercury determination

| | F1 | F2 | F3 | F4 |
|---|---|---|---|---|
| Curve's equation | y = 300.23x + 199.62 | y = 252.95 + 66.41 | y = 323.64 - 419.7 | 326.78x + 43.58 |
| Coefficient of determination ($R^2$) | 0.999 | 0.999 | 0.995 | 0.999 |
| LD | 0.2 $\mu g.L^{-1}$ | 0.3 $\mu g.L^{-1}$ | 1.3 $\mu g.L^{-1}$ | 0.08 ng |
| Linear range of work | 0.2 to 80 $\mu g.L^{-1}$ | 0.3 to 80 $\mu g.L^{-1}$ | 1.3 to 80 $\mu g.L^{-1}$ | 0.08 to 25.23 ng |

Concentrations in F1, F2 and F3 were determined by cold vapor atomic absorption spectroscopy (CVAAS).
Concentrations in F4 were determined by thermal desorption atomic absorption spectroscopy (TDAAS).

**Table S2** - EDS results in % weight of each element.

| Point | O | F | Mg | Al | Si | P | K | Ti | Fe | Zr | Sn | La | Ce | Hg | Th |
|---|---|---|---|---|---|---|---|---|---|---|---|---|---|---|---|
| **1** | **40.31** | **0** | **0** | **1.81** | **15.13** | **4.88** | **0** | **0.67** | **4.69** | **29.02** | **0** | **0** | **0.51** | **1.51** | **1.49** |
| 2 | 37.69 | 0 | 0 | 5.98 | 14.31 | 11.12 | 0 | 0.61 | 16.41 | 9.11 | 0 | 0 | 2.06 | 0 | 2.72 |
| 3 | 32.63 | 0 | 0 | 0.25 | 14.33 | 2.14 | 0 | 0.75 | 3.49 | 36.88 | 0.65 | 3.35 | 0.81 | 0 | 4.7 |
| 4 | 0 | 0 | 0 | 0 | 11.62 | 3.45 | 0 | 10.28 | 49.17 | 25.48 | 0 | 0 | 0 | 0 | 0 |
| 5 | 27.74 | 0 | 0 | 0 | 22.83 | 3.29 | 0 | 2.52 | 0 | 43.62 | 0 | 0 | 0 | 0 | 0 |
| 6 | 30.18 | 0 | 0 | 0 | 1.17 | 0 | 0 | 31.8 | 30.4 | 0 | 0.59 | 2.19 | 3.67 | 0 | 0 |
| **7** | **41.46** | **0** | **0** | **21.12** | **14.68** | **1.58** | **0** | **0.6** | **15.58** | **3.18** | **0** | **0.87** | **0** | **0.94** | **0** |
| **8** | **42.93** | **0** | **0** | **3.76** | **11.97** | **2.86** | **0** | **0** | **14.38** | **19.53** | **0** | **0** | **2.92** | **1.64** | **0** |
| **9** | **33.89** | **0** | **0** | **0.31** | **13.34** | **3.77** | **0** | **0.55** | **2.1** | **45.17** | **0** | **0** | **0.5** | **0.29** | **0.08** |
| 10 | 38.01 | 0 | 0 | 1.38 | 2.25 | 11.55 | 0 | 1.57 | 17.05 | 0 | 0 | 2.71 | 20.35 | 0 | 5.12 |
| 11 | 38.57 | 0 | 0 | 3.42 | 12.64 | 1.63 | 0 | 0.77 | 18.17 | 21.99 | 0 | 1.51 | 1.31 | 0 | 0 |
| 12 | 38.97 | 0 | 0 | 0.33 | 12.08 | 2.77 | 0 | 0 | 2.09 | 43.76 | 0 | 0 | 0 | 0 | 0 |
| 13 | 32.48 | 0 | 0 | 1.14 | 2.87 | 12.36 | 0 | 0 | 6.1 | 0 | 1.33 | 12.67 | 28.23 | 0 | 2.82 |

| | | | | | | | | | | | | | | | |
|---|---|---|---|---|---|---|---|---|---|---|---|---|---|---|---|
| 14 | 37.19 | 0 | 0 | 0.53 | 0.68 | 14.68 | 0 | 0 | 2.76 | 0 | 0.63 | 8.88 | 29.38 | 0 | 5.26 |
| **15** | **26.52** | **0** | **0** | **0.66** | **1.51** | **0.44** | **0** | **0** | **2.35** | **0.15** | **65.21** | **0** | **0** | **0.71** | **2.45** |
| **16** | **39.42** | **0** | **0** | **3.91** | **15.09** | **4.2** | **0** | **0** | **9.39** | **25.93** | **0** | **0** | **0** | **2.06** | **0** |
| 17 | 25.14 | 0 | 0 | 0.27 | 15.39 | 3.29 | 0 | 0.77 | 6.28 | 42.61 | 0 | 3.61 | 2.64 | 0 | 0 |
| 18 | 27.46 | 1.55 | 0 | 1.39 | 0.31 | 0.32 | 0 | 0.33 | 65.58 | 0 | 1.35 | 1.7 | 0 | 0 | 0 |
| **19** | **36.08** | **0** | **0** | **0.97** | **1.76** | **0.45** | **0** | **0.62** | **54.07** | **1.54** | **0.95** | **3.11** | **0** | **0.48** | **0** |
| **20** | **40.26** | **0** | **0** | **0** | **11.87** | **2.43** | **0** | **0.27** | **4.35** | **36.84** | **2.27** | **0** | **0** | **1.72** | **0** |
| **21** | **25.13** | **0** | **0** | **0.08** | **0.06** | **0** | **0** | **0** | **72.64** | **0** | **0.44** | **0** | **0** | **0.87** | **0.78** |
| **22** | **38.21** | **0** | **0** | **0.78** | **12.86** | **3.68** | **0** | **1.31** | **9.52** | **32.02** | **0** | **0** | **0** | **1.47** | **0.15** |
| **23** | **27.49** | **0** | **0** | **0.29** | **15.84** | **4.53** | **0** | **0** | **1.68** | **48.59** | **0.82** | **0** | **0** | **0.76** | **0** |
| 24 | 36.41 | 0 | 0 | 0.75 | 20.56 | 2.66 | 0 | 2.5 | 6.83 | 29.98 | 0 | 0 | 0 | 0 | 0.31 |
| **25** | **52.68** | **0** | **0** | **0.7** | **41.04** | **0.34** | **0** | **0.59** | **0.86** | **0** | **1.1** | **0** | **0** | **1.59** | **1.1** |
| 26 | 50.79 | 0 | 3.08 | 10.9 | 20.32 | 0.05 | 4.79 | 0.77 | 4.11 | 0 | 1.76 | 0 | 2.28 | 0 | 1.17 |
| 27 | 49.61 | 0 | 2.81 | 9.35 | 22.07 | 0 | 4.04 | 0.37 | 9.79 | 0.11 | 1.39 | 0.46 | 0 | 0 | 0 |
| **28** | **47.94** | **0** | **0** | **0** | **39.23** | **0.41** | **0** | **0.11** | **7.03** | **1.98** | **0.46** | **0.72** | **0** | **1.62** | **0.51** |

Rows in bold are the points containing Hg in its chemical composition. Points 2, 10, 13 and 14 have chemical compositions close to that of monazite.

**Table S3** - Major elements concentration in the ASGM tailings sample

| | **Concentration (%)** |
|---|---|
| Fe | 21.56[1] |
| Zr | 13.92[1] |
| Si | 12.71[1] |
| Ti | 6.33[1] |
| Al | 0.81[1] |
| P | 0.60[1] |
| Mn | 0.32[1] |
| Ca | 0.21[1] |
| Mg | 0.16[1] |
| K | 0.53[2] |
| Na | 0.07[2] |

1 – determined by ICP-OES; 2 – determined by ICP-MS.

**Table S4** - Elements concentration in the first three sequential extraction procedure steps

| | **F1 ($mg.kg^{-1}$)** | **F2 ($mg.kg^{-1}$)** | **F3 ($mg.kg^{-1}$)** |
|---|---|---|---|
| Na | 13 ± 1 | 7 ± 1 | 2.1 ± 0.7 |
| K | 206 ± 7 | 37 ± 2 | <LD |
| Al | 125 ± 4 | 512 ± 6 | 385 ± 9 |
| P | 12 ± 3 | 319 ± 2 | 146 ± 7 |

| | | | |
|---|---|---|---|
| Si | 119.5 ± 0.3 | 192 ± 4 | 164 ± 5 |
| Mg | 51 ± 2 | 209 ± 9 | 134 ± 3 |
| Mn | 65.3 ± 0.4 | 369 ± 3 | 62.0 ± 0.4 |
| Ti | <LD | 49.0 ± 0.2 | 154 ± 1 |
| Fe | 36.9 ± 0.2 | 7987 ± 320 | 22185 ± 107 |
| Zr | <LD | 36.3 ± 0.2 | 10.4 ± 0.1 |

Concentrations are presented accompanied by their corresponding confidence interval at a 95% confidence level.
